# Nondestructive Detection of Moisture Content in Palm Oil by Using Portable Vibrational Spectroscopy and Optimal Prediction Algorithms

**DOI:** 10.1155/2023/3364720

**Published:** 2023-01-31

**Authors:** Ernest Teye, Charles L. Y. Amuah, Tai-Sheng Yeh, Regina Nyorkeh

**Affiliations:** ^1^Department of Agricultural Engineering, School of Agriculture, College of Agriculture and Natural Sciences, University of Cape Coast, Cape Coast, Ghana; ^2^Department of Physics, Laser and Fibre Optics Centre, School of Physical Sciences, University of Cape Coast, Cape Coast, Ghana; ^3^Department of Food Science and Nutrition, Meiho University, Neipu Township, Taiwan

## Abstract

Rapid and nondestructive measurement of moisture content in crude palm oil is essential for promoting the shelf-stability and quality. In this research, micro NIR spectrometer coupled with a multivariate calibration model was used to collect and analyse fingerprinted information from palm oil samples at different moisture contents. Several preprocessing methods such as standard normal variant (SNV), multiplicative scatter correction (MSC), Savitzky–Golay first derivative (SGD1), Savitzky–Golay second derivative (SGD2) together with partial least square (PLS) regression techniques, full PLS, interval PLS (iPLS), synergy interval PLS (SiPLS), genetic algorithm PLS (GAPLS), and successive projection algorithm PLS (SPA-PLS) were comparatively employed to construct an optimum quantitative prediction model for moisture content in crude palm oil. The models were evaluated according to the coefficient of determination and root mean square error in calibration (Rc and RMSEC) and prediction (Rp and RMSEC) set, respectively. The model SGD1 + SiPLS was the optimal novel algorithm obtained among the others with the performance of Rc = 0.968 and RMSEC = 0.468 in the calibration set and Rp = 0.956 and RMSEP = 0.361 in the prediction set. The results showed that rapid and nondestructive determination of moisture content in palm oil is feasible and this would go a long way to facilitating quality control of crude palm oil.

## 1. Introduction

Palm oil is the most consumed edible vegetable oil in the world with various applications in food products including the production of margarine, ice creams, crackers, chocolates, and fried foods, among others [1] Demand for palm oil continues to grow steadily worldwide as global production falls short of supply at 70 million metric tonnes since 2017 [2]. Generally, palm oil is rich in carotenoids and other very important nutritional phytonutrients such as vitamin E components (tocopherols and tocotrienols) and it is known to provide health-beneficial properties.

During extraction and processing of crude palm oil, the moisture content is monitored until it gets to its final required state as this determines the quality during storage. Also, the moisture content is among the parameters that dictate the price. Research has shown that the moisture content of palm oil increases water activity and this further leads to high hydrolysis and is a possible cause of a steady rise in free fatty acid values during storage [1]. Other studies have shown that moisture content and amount of free fatty acid (FFA) are the important quality parameters of palm oil [3]. High moisture content causes rancidity, Aspergillus Niger, and Mucor species growth in edible oils with high moisture content [4]. Recently, high moisture contents had been reported in palm oil and raised concerns for storage stability and spoilage [5]. A cost-effective method for moisture determination of palm oil is therefore urgently needed. Therefore, measuring the moisture content of crude palm oil during processing and storage is vital to ensuring quality and maintaining storage life.

Various techniques had been employed previously to detect moisture in edible oil. Traditionally, the moisture content is determined by the oven drying method; the Karl Fisher method has been employed frequently for the determination of moisture content, and this method uses a complicated titrator, expensive chemicals, and time-consuming procedures. Also, others use different methods such as over dry methods as carried out by others [6], microwave six-port reflectometer [7], and the pure microwave method and titration method among others. However, these methods are time-consuming, cumbersome, labour intensive and require laboratory infrastructure, cannot be used on-site, and require skilled personnel. Furthermore, headspace GC had also been proposed for the determination of moisture in edible oil, but the instrument demands higher maintenance costs [8].

Therefore, processors together with quality control officers require rapid and nondestructive determination of moisture content in palm oil. NIR spectroscopy offers a possible solution for the rapid determination of palm oil moisture content. This technique has been used for assessing the quality parameter of other edible oils [9, 10]. Thus, spectroscopic techniques have proven to turn out results quickly without using expensive chemicals. Moisture content in edible oil had been determined by FTIR with transmission measurement through NaCl window [11], dry solvent extraction [12–14], reaction method [15, 16], and by the application of infrared transparent PTFE membranes for transmission measurement [17]. Moisture in olive oil had been determined by the NIR spectra method [18]. Also, the univariate NIR method was used to improve the speed of measurement of moisture content with disposable glass tubes and PLS multivariate data analysis [19]. The recent miniaturization of the NIR spectrometer has been found useful in different food analysis applications [20–22]. Other previous studies have been reported on the use of desktop FTIR or NIR spectrometer for moisture content in edible oil. It will therefore be of great interest to see whether a portable NIR spectrometer could perform a similar task as the desktop version. Herein, the present study employs pocket-size NIR spectrometer for rapid determination of moisture content in palm oil. Up until now, little or no studies have investigated the use of portable NIR spectroscopy for on-site detection of palm oil moisture content in developing countries. Furthermore, proper preprocessing of spectra data is known to have an impact on the multivariate data analysis [23–25]; hence, in this study, different signal preprocessing methods would be employed comparatively to develop a robust optimal method. Moreso, the variable selection method would be an additional advantage of this study because research in other fields has shown that, it improves the performance of the regression model [26–28]. Thus, the variable selection methods, such as interval PLS (iPLS), synergy interval PLS (SiPLS), genetic algorithm PLS (GA-PLS), and successive projections algorithm (SPA-PLS), were used and compared to find the best variable selection method for PLS.

## 2. Materials and Methods

### 2.1. Sample Collection

Palm oil samples were collected from five major palm oil-producing regions in Ghana at different moisture contents; others were specifically collected directly from factories in central and western regions of Ghana. All the palm oil samples were at different levels of moisture content. A total of 150 samples were collected into smaller 250 ml bottles and transported into the laboratory in the School of Agriculture Technology Centre.

### 2.2. Spectral Collections

In the laboratory, the samples were scanned individually and the spectrum was collected using a small pocket-sized NIR spectrometer (SCIO™) in the range of 740–1070 nm in a 1-nm resolution for spectra data recording assisted by a smartphone (Nokia 6). For each sample, the palm oil was poured into a Petri dish and scanned three times after rotating the sample cup as carried out by others [3]. The entire process was carried out at an ambient temperature of 31°C with a steady state of humidity at the laboratory of the Food fraud and safety centre of the School of Agriculture, University of Cape Coast. All the samples were analyzed in triplicate and the spectra were averaged to provide a mean spectrum as the original spectrum of the sample used.

### 2.3. Moisture Content Determination

The moisture content of all the samples was carried out using the standard method according to the standard used by other authors [6]. The moisture contents of the samples were carried out in triplicate and average to represent one sample.

### 2.4. Chemometric Analysis

To analyse the spectra fingerprint, the data recordings stored in the cloud were downloaded onto the computer and imported into chemometric software in MATLAB (2021a; MathWorks Inc., USA) using windows 10 Basic for all data processing. The fingerprinted information was modelled and compared using different algorithms to determine the optimum technique for determining moisture content in palm oil.

### 2.5. Pretreatment Methods

In this study, several mathematical transformational techniques compared with raw (no treatment) were used to improve spectra fingerprinted data. These techniques used include standard normal variant (SNV), multiplicative scatter correction (MSC), first derivative (*D*1), and second derivative (*D*2). These preprocessing treatments have their unique strengths and weaknesses in spectra fingerprint; for more information on their theoretical background, refer to other authors [29–31]. In this study, Savitzky–Golay smoothing was performed on the derivative spectra treatments (first and second; SGD1 and SGD2) to eliminate noise which is known to be a drawback of derivative methods [31]. All these pretreatment techniques were carried out to improve the correlation between spectra fingerprint and chemical composition of interest as in the case of moisture content in our study.

### 2.6. Full and Variable Selection Algorithms

The study also employed full and variable spectra selection quantitative prediction techniques by using partial least square regression (PLSR), interval PLS (iPLSR), synergy interval PLS (SiPLS), genetic algorithm PLS (GaPLS), and successive projection algorithm PLS (SPA-PLS). For more information on the theories of the regression methods used kindly refer to other authors [30, 32]. The performances of the algorithms used were compared and evaluated in terms of correlation determination of calibration (Rc), correlation of prediction determination (Rp), root mean square error of calibration (RMSEC), and root mean squared error of prediction (RMSEP).

## 3. Results and Discussion

### 3.1. Moisture Content in Palm Oil

The 409 samples of palm oil with unique moisture contents were used in this study, and the values cover all the range of moisture content of palm oil during processing in the factory as well as in the various markets in Ghana as seen in Table 1. The moisture content of the various samples ranges from 0.060 to 7.220%. From the table, it could also be seen that the palm oil samples used had a wide range of values to cover the entire moisture content levels observed during processing and storage. This is particularly useful as it makes the model robust.

### 3.2. Data Preprocessing and Splitting

To select a set of representative objects for calibration/prediction set in the PLS models, the Kennard–Stone algorithm was employed [33].

### 3.3. Spectra Examination

**Figure 1:**
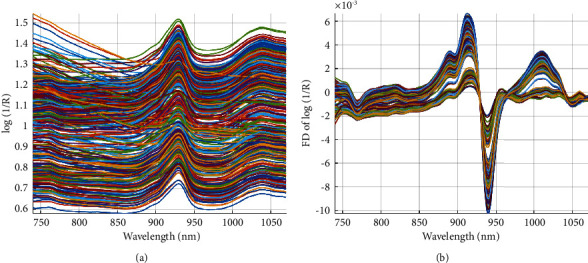
Raw (a) and SGD1 (b) preprocessed spectra of crude palm oil.

The spectra profiles of the palm oil samples were mathematically pretreated by different techniques and their unique fingerprints were observed in the study as shown in Figure 1(a). It is well known that each pretreatment method showed unique properties that contribute to enhancing the performance of multivariate algorithms. Among the different pretreatment used, SNV, MSC, SGD1, and SGD2 spectra are not significantly different from each other. Major peaks were seen in the wavelength range of 750–800 nm, 850–900 nm, 910–950 nm, and 1025–1050 nm. Thus, these wavelengths could be responsible for O-H deformation and O-H stretching which corresponds to water. These peaks vary from one pretreatment to the other. Most especially, the peaks were more pronounced when Savitzky–Golay smoothing first derivative spectra pre-treatment (Figure 1(b)) was employed, and this is a typical characteristic of derivative pretreatments. Also, these spectra wavelengths are made up of carbonyl group; C-H stretch and C-H deformation correspond to phytochemicals in palm oil.

### 3.4. Effect of Pretreatment on PLS Regression

**Figure 2:**
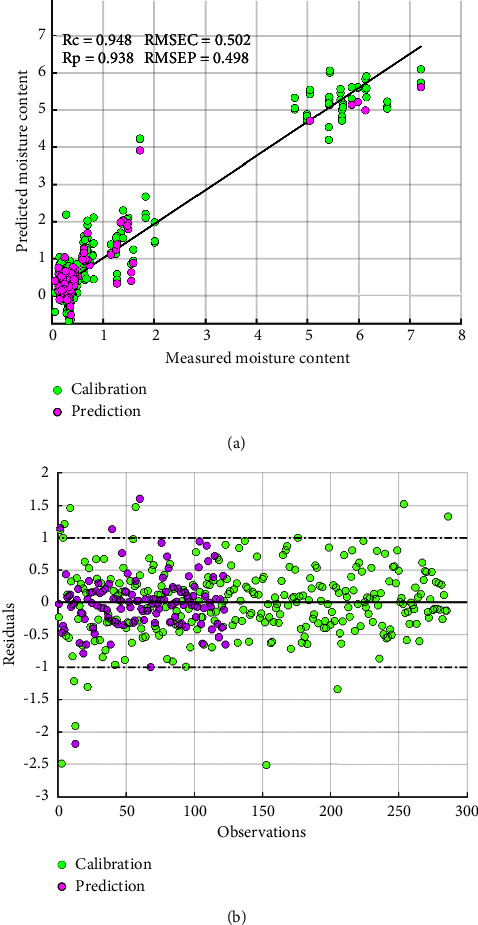
SGD1-PLS score plot of measured versus predicted moisture content (a) and residuals versus samples (b).

The performance of PLS regression to the determined moisture content in palm oil was modelled with the help of different pretreatments. The original spectral profile obtained contains information related to the chemical composition of the samples, as well as irrelevant interference data such as baseline drift, sample physical properties, background, and noise [34]. These weaknesses in the data directly affect the accuracy of the final outcome. Hence, to improve the modeling efficiency of the moisture content, SNV, MSC, SGD1, and SGD2 were used to preprocess the original spectral data. The unique profile and results for each preprocessing treatment are shown in Table 2. From this table, it could be seen, all the pretreatment methods had a different impact on the final results. More importantly, Savitzky–Golay smoothing first derivative (SGD1) spectra pretreatment performed better than all the others, with an increased performance of *R* = 0.948 and RMSEP = 0.586 in the prediction set. As seen in Figure 2, the residuals were randomly also scattered about their mean value. Also, the SGD1 preprocessing method made the best impact on the performance of the model in this study, with an improved prediction efficiency. However, PLS uses a full spectra range that contains both useful and redundant information. Therefore, the modeling was further optimized by employing other interval spectral selection algorithms in this study.

### 3.5. Interval Selection PLS Regression Algorithms

**Figure 3:**
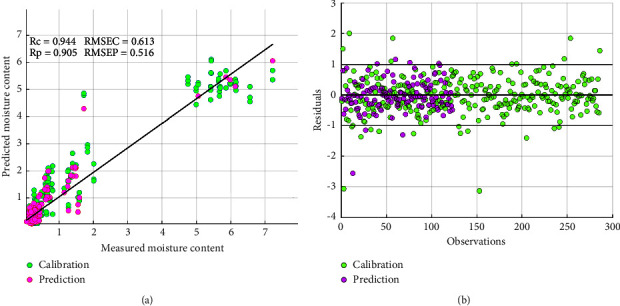
SGD1-iPLS score plot of measured versus predicted moisture content (a) and residuals versus samples (b).

**Figure 4:**
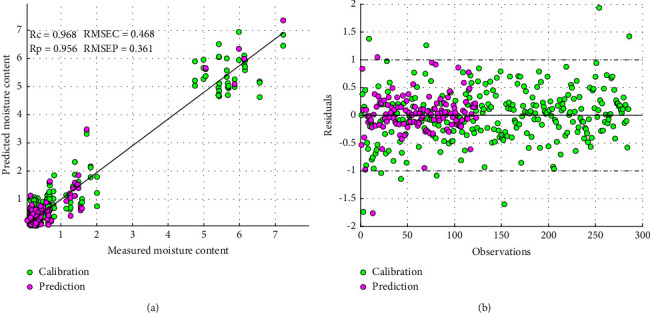
SGD1-SiPLS score plot of measured versus predicted moisture content (a) and residuals versus samples (b).

Norgaard and other researchers proposed interval selection PLS (iPLS) and synergy interval selection PLS (SiPLS) to overcome the weaknesses and challenges of full PLS regression in spectra data analysis [35]. In this study, iPLS was attempted to optimize the results and further prove the strength of interval spectra selection. From Table 3, it could be observed that iPLS was optimized with 13 best intervals with a performance of Rc = 0.944 and Rp = 0.905. This outcome showed a slight similarity to the full PLS regression results in the calibration set but less in the prediction set. This could be explained that iPLS actually solved the weaknesses of full PLS by selecting only one maximum region that corresponded to moisture content to calibrate the PLS model. However, selecting only one wavelength interval could lead to leaving out other equally important spectra information; therefore, this could influence the performance of the model [36]. Also, as seen in Figure 3, the residuals are distributed about the mean value which is good. On the other hand, SiPLS which solves the shortcomings of iPLS was also comparatively used. From Table 4, it could be observed that SiPLS showed its unique superiority with the model performance of Rc = 0.968 and Rp = 0.956 (Figure 4(a)). The best optimal interval of 986–1002 nm and 1003–1019 nm at 5 latent variables were selected. These optimal spectra intervals selected by SiPLS corresponded to various absorption bands that could be related to moisture content and water activities in palm oil. As seen from Figure 4(b), the residual was randomly distributed about the mean value and comparatively and satisfactorily close to zero (0) thus low bias [37].

### 3.6. General Discussion

The optimal performance of a micro-NIR spectrometer coupled with different multivariate regression models was comparatively studied. As seen in Table 5, it was observed that different regression models performed differently for the moisture content model in palm oil. At a full range of 740–1070 nm, the PLS model performed at Rp = 0.943 which is fairly good; however, an attempt to improve the results by using other algorithms revealed that iPLS showed slightly similar performance to PLS while SiPLS performed better than iPLS and full PLS. It could be explained that SiPLS selected only relevant spectra information and combined them as in the case of this study (986–1002 nm, and 1003–1019 nm) to calibrate the PLS model so that much useful information that corresponded to the moisture content in the palm oil would be included in the universal model. On the other hand, the comparatively less performance of PLS, iPLS, and GAPLS than that of SiPLS could be a result of weaknesses in full PLS range spectra where the entire spectra are mixed with useful and redundant information that could have influenced the results. For iPLS, the selection of only one interval could result in leaving out other equally important spectra information that could have improved the results. While for GAPLS, the limitation was the fact that when spectra intensities are measured at a very large number of wavelengths, the search domain increases correspondingly, and therefore, the detection of the relevant regions is much more difficult and hindered [38]. Also, the successive projection algorithm (SPA) regression used, performed quite well; however, it showed overfitting as the calibration set had significantly lower than the prediction set. However, the comparatively better prediction results may be ascribed to the removal of uninformative variables from the modeling process [32]. Also, SPA is known to select a subset of variables with small multicollinearity and suitable prediction power [32].

## 4. Conclusion

Moisture content in palm oil has been determined by using a micro-NIR spectrometer together with multivariate algorithms. Generally, the findings revealed that Savitzky–Golay first derivative transformation techniques together with the partial least square regression (PLSR) model, specifically synergy interval partial least square (Si-PLS), could be used to develop a prediction equation from the spectra data set to quantify moisture content in palm oil samples at a favourable coefficient of prediction above 0.94. Among the models used (PLSR, i-PLSR, Si-PLSR, GA-PLSR, and SPA-PLSR), SGD1 together with the Si-PLS model was superior to all with the model performance of Rc = 0.968 and RMSEC = 0.468 in the calibration set and Rp = 0.956 and RMSEP = 0.361 in the prediction set. The results showed that rapid and nondestructive determination of moisture content in palm oil is feasible and this would go a long way to facilitating quality control of crude palm oil. The study provides feasibility, and further work is needed to include wide samples from different locations and factory settings to make the technique universal and robust. Furthermore, this research provides the potential of incorporating portable NIR spectrometers into a smartphone for use by rural processors and quality control officers.

## Figures and Tables

**Table 1:** Reference measurement of moisture content (Mc) and statistic data.

Parameter	Subset	Number	Max	Min	Mean	Std
Mc (%)	Calibration	286	7.220	0.060	1.212	1.879
	Prediction	123	6.130	0.060	0.670	1.112

**Table 2:** Effect of spectra pre-treatment on PLS regression.

Pretreatment	LV	Rc	RMSEC	Rp	RMSEP
Raw	2	0.897	0.893	0.941	0.500
SNV	3	0.931	0.566	0.912	0.415
MSC	3	0.818	1.318	0.733	0.612
SGD1^1^	3	0.948	0.586	0.938	0.574
SGD2^2^	2	0.941	0.599	0.932	0.553

**Table 3:** SGD1-iPLS result for different numbers of intervals.

Number of intervals	Best interval	Rc	RMSEC	Rp	RMSEP
10	7	0.909	0.700	0.844	0.634
11	7	0.937	0.650	0.901	0.527
12	8	0.943	0.584	0.899	0.513
**13**	9	0.944	0.614	0.905	0.516
14	9	0.945	0.610	0.879	0.581
15	10	0.930	0.643	0.840	0.620
16	11	0.926	0.708	0.838	0.669
17	10	0.925	0.706	0.856	0.646
18	12	0.921	0.674	0.855	0.658
19	13	0.929	0.688	0.864	0.638
20	13	0.914	0.760	0.827	0.705

Best results.

**Table 4:** SGD1-Si-PLS result for different number of intervals.

Number of intervals	Best interval	Rc	RMSEC	Rp	RMSEP
10	1 2 7 10	0.956	0.560	0.925	0.460
11	1 3 8 10	0.956	0.549	0.933	0.451
12	2 4 8 12	0.956	0.553	0.920	0.472
13	7 8	0.965	0.484	0.952	0.347
14	2 7 10 13	0.957	0.564	0.931	0.434
15	8 9	0.961	0.514	0.952	0.359
16	2 8 11 13	0.958	0.568	0.930	0.448
17	13 14	0.963	0.501	0.935	0.423
18	10 11	0.964	0.475	0.951	0.343
19	15 16	0.968	0.468	0.956	0.361
20	10 12	0.964	0.492	0.948	0.392

**Table 5:** Optimum comparison of model performance for different variable selection techniques.

Method	Selected wavelength (nm)	Rc	RMSEC	Rp	RMSEP
PLS	740–1070	0.943	0.582	0.938	0.474
i-PLS	943–958	0.944	0.614	0.905	0.516
Si-PLS	986–1002, 1003–1019	0.968	0.468	0.956	0.361
GA-PLS	740–1070	0.952	0.553	0.950	0.387
SPA-PLS	768, 778, 816, 932, 953, 1009	0.957	0.558	0.941	0.493

## Data Availability

The data used to support the findings of this study are available from the corresponding author upon request.

## Additional Points

Determination of moisture content in palm oil is a vital activity that ensures the overall quality of the oil. Especially during the extraction and processing of palm oil, rapid monitoring of moisture content until it gets to the final required state is a key determinant of shelf-life. However, the conventional analytical methods for the determination of the moisture content of palm oil are tedious and time-wasting. This research work provides a practical and efficient alternative method for rapid, accurate, and nondestructive determination of moisture content in palm oil using portable NIR spectroscopy combined with a regression algorithm.
